# Supplementary material for: Offline ventral subiculum-ventral striatum serial communication is required for spatial memory consolidation
Source: Nat Commun. 2019 Dec 16;10:5721. doi: 10.1038/s41467-019-13703-3 (PMC6915753; doi:10.1038/s41467-019-13703-3)
Supplement: Supplementary file 1 — Supplementary Information [file 41467_2019_13703_MOESM1_ESM.pdf]

## SUPPLEMENTARY INFORMATION

# OFFLINE VENTRAL SUBICULUM-VENTRAL STRIATUM SERIAL COMMUNICATION IS REQUIRED FOR SPATIAL MEMORY CONSOLIDATION

Torromino et al.,

## SUPPLEMENTARY METHODS

### Behavioral procedures

For the assessment of possible non specific effects of $hM4D_i$ activation in the VS-projecting subicular terminals mice were tested in the elevated plus maze, in the open field and for novelty exploration. To determine possible non specific effects of intra VS CNO administrations alone an independent group of mice was tested in the open field.

The elevated plus maze (EPM) apparatus was made of black Plexiglas and consisted of two open and two closed arms separated by a center square platform (5 x 5 cm). Each arm was 5 cm wide and 35 cm long. The maze was positioned 50 cm from the ground. At the beginning of the procedure, mice were isolated in a waiting cage for 15 min, next they were administrated with CNO 10 mM or vehicle (DMSO/PBS) in the VS and placed back in their waiting cage for additional 15 minutes. The task consisted in a single trial test: each mouse was placed in the center platform facing the open arm and allowed to freely explore for 5 minutes. At the end of the task, mice returned in their waiting cages for 20 minutes before being tested in the open field. Between consecutive tests, the apparatus was cleaned with 70% ethanol solution. The operational criterion for entry was the whole body with four paws inside the arm. The percentage of time spent in each arm (time spent in an arm/total testing time x 100) was manually scored from the recorded videotapes, while the total distance travelled was automatically scored by using Anymaze software (ANY-maze, Stoelting, USA).

The open field apparatus consisted of a rectangular open field (60 x 48 cm) made of plastic material. The apparatus was placed in a soundproof cubicle and illuminated by a white light (60W) located on the ceiling. Animals were placed in the empty arena and left free to explore for 5 minutes. At the end of the procedure, animals returned in their home cage for additional 5 minutes before being tested for object novelty. The total distance travelled was automatically quantified by using Anymaze software (ANY-maze, Stoelting, USA).

For the intra VS CNO control experiments, the apparatus consisted in a squared arena (40 x 40 cm). At the beginning of the procedure, mice were isolated in a waiting cage for 15 min, then they were

administrated with CNO 10 mM or vehicle (DMSO/PBS) in the VS and they were placed back in their waiting cage for additional 15 minutes. The task consisted in a single trial test, in which they were introduced in the empty arena and left free to explore for 1h. The floor of the arena was divided in 16 equal quadrants for the analysis of locomotor, exploratory activity and anxiety. Mice behavior was scored *a posteriori* with Anymaze Software (ANY-maze, Stoelting, USA).

For the object novelty experiment mice were introduced in the arena facing the wall and left free to explore for 5 minutes. 10 cm away from the opposite wall was placed an object consisting of a black iron angle with four holes for each side (5 cm diameter, 7 cm high) attached to a transparent Plexiglas base. The latency to approach the object and the time spent exploring the object was manually scored *a posteriori* using Anymaze software (ANY-maze, Stoelting, USA).

## Immunofluorescence

For Venus reporter and HA-tagged hM4D$_i$ visualization experiment mice injected with AAV-Syn::Venus-2A-HA-hM4D$_i$ were deeply anaesthetised with an overdose of anaesthetic and transcardiacally perfused as described for the FG-immunofluorescence procedure. Brains were post-fixed for 4hrs in PFA 4% and transferred to a 30% sucrose solution for 48hrs. 40 µm coronal slices were collected with a microtome (Leica Microsystem, Germany) and kept in PBS and sodium azide (0.05%) solution. Antigen retrieval for HA-detection was obtained by heating VS-slices in sodium citrate buffer solution (10 mM, pH 6.0) in a dry-bath at 100°C for 8 min. Slices were then left at room temperature for 30 min before 1h incubation with PBS Triton 0.3%. They were then incubated for 1h in a blocking solution with PBS Triton 0.3% and NGS 5% at room temperature. Successively, slices were incubated overnight at 4°C with the anti-HA primary antibody raised in rabbit (1:500; HA-Tag, Cell Signaling Technology) diluted in 5% NGS and PBST 0.3%. After incubation, slices were washed 10 min in PBS for three times and then incubated with the secondary antibody for 90 min (1:300, Rhodamine Red™-X-conjugated goat anti-rabbit, 111-295-144, ImmunoResearch, USA). After four additional washes in PBS for 10 min, slices were mounted on gelatin-coated slides and coverslipped with Vectashield Hard Set (Vector Laboratories, Inc.).

Images were acquired at the Olympus iX83-FV1200 confocal laser scanning microscope with a 10x NA

0.40 objective, 473nm laser/EYFP setting for Venus detection and 559nm laser/Rhodamine Red setting for HA detection. Single images of 1600x1600 pixels were stitched together in a mosaic view with the Multi Area Viewer tool (Olympus Fluoview 4.2). For HA three alternate VS slices per four representative mice were analyzed. Consecutive to the previous slices were analyzed for Venus detection. Images were converted in binary and area of the extension of Venus and HA on respective consecutive slices was outlined and determined with the use of ImageJ Software (NIH Image, USA). The percentage of the overlapping area of Venus signal in respect to HA diffusion was then calculated.

**Injection placement verification and Venus protein diffusion**

After the end of the behavioural procedure, the correct positioning of the injection for pharmacological and chemogenetics experiments, as well as virus expression, were verified.

For pharmacological experiments, mice were sacrificed with the use of isofluorane followed by cerebral dislocation. Brains were dissected and put in a 4% paraformaldehyde solution in PBS for 48h. With the use of a freezing microtome (Leica Microsystem, Germany), they were sliced at 90 μm. Serial slices were collected on gelatin-coated slides and stained with Cresyl Violet (Sigma-Aldrich, Italy). They were then analysed with the use of a stereomicroscope and the most ventral point of the tip of the injector was identified. Illustrations of coronal sections from single animals are represented for each pharmacology experiment.

For Venus protein detection, mice were first perfused as described above, and 40 μm vSUB and VS sections were cut at a freezing microtome, mounted on gelatin-coated slides, coverslipped with Vectashield Hard Set (Vector Laboratories, Inc.). To verify the diffusion of the viral vector, Venus protein fluorescence was detected under a confocal microscope (Leica DMI6000) for both vSUB and VS (488 nm laser) and the sections with the most diffused signal was acquired. Microphotographs of each hemisphere were taken at 5x magnification (1024x1024 pixel). Illustration of Venus diffusion for each mouse are reported for both the vSUB and the VS. The diffusion of Venus expression was schematized for each subject on coronal sections of the mouse brain atlas (Franklin and Paxinos, 1997) with the use of Adobe Illustrator (Adobe Systems Incorporated, USA). Total VS area and Venus diffusion area were

outlined and determined for each section with the use of ImageJ (NIH). Only mice showing Venus diffusion in at least the 70% of total VS extension were included in statistics.

**Statistics**

All data are represented as mean ± standard error of the mean (SEM). For statistical analysis Statview Software (Scientific Computing, North Carolina), Statistica Software (Dell Software, Oklahoma) and GraphPad Prism were used. Group differences were considered statistically significant when $p \leq 0.05$.

To compare naïve, cue and spatial trained animals in the fos/FG experiment a one-way ANOVA with training (three levels: naïve, cue, spatial) as between-groups factor was used for each variable (FG/$mm^2$, fos/$mm^2$ and fos/FG co-localization). Fisher's PLSD was used for *post-hoc* comparison.

For the analysis of the training phase in the MWM, distance travelled to the platform was analysed using a two-way, repeated measure ANOVA with post-training treatment (two levels: PBS, AP-5) as between-groups factor and sessions (six levels: session 1 to 6) as repeated measures. When the interaction between factors was not significant, a one-way, repeated measures ANOVA with sessions (six levels: session 1 to 6) as repeated measures was used on each group independently.

For the probe test in the MWM experiments, distance travelled in each quadrant was analysed using a two-way, repeated measure ANOVA with post-training treatment (two levels: PBS, AP-5) as between-groups factor and quadrants (four levels: target, right, opposite, left) as repeated measures. When no significant interaction between factors was revealed, data were analysed independently in each group through a one-way repeated measures ANOVA (four levels: target, right, opposite, left). Tukey Honestly Significant Difference (HSD) was used as *post-hoc* comparison. First bearing in the probe trial was analyzed using an unpaired student's t-test.

For the ODT experiment, total sector crossings were analysed for the S1 with one-way ANOVA (two levels: PBS, AP-5). The total exploration was considered for session S2 to S4 and analysed with a two-way, repeated measures ANOVA with sessions (three levels: S2 to S4) as repeated measures and post-training treatment (two levels: PBS, AP-5) as between-groups factor. Only animals showing exploration decreased from S2 to S4 where included in statistics. As an index of object discrimination, exploration

for displaced (DO) and non-displaced objects (NDO) at S5 and S4 was compared. A three-way repeated measures ANOVA with sessions as repeated measures (two levels: S4, S5), post-training treatment (two levels: PBS, AP-5) and object category (two levels: DO, NDO) as between-groups factors was used. Fisher's PLSD *post hoc* analysis was then used. Leaning, rearing and grooming were scored at S5 and analysed with one-way ANOVA (two levels: PBS, AP-5).

For open field experiment total distance travelled, percentage time spent in the peripheral and central quadrants of the arena, leaning, rearing and grooming were analysed using a one-way ANOVA with pre-training treatment (two levels: PBS, AP-5) as between factor. For the EPM, the open field, and the novelty experiments vehicle and CNO administered groups were compared using an unpaired Student's t-test.

For acute slice recordings values significance of differences for sEPSCs was assessed by Student's paired t test. Shapiro-Wilk test was performed to assess normal distribution.

Dendritic spine density was analysed with a two-way ANOVA, with training (two levels: cue, spatial) and post-training treatment (two levels: PBS, AP-5) as between-groups factors. Tukey HSD was used for *post-hoc* comparison.

## Supplementary References

1. Franklin BJ, & Paxinos G. *The mouse brain in stereotaxic coordinates*. San Diego: Academic Press 1997).

2. Ferretti, V. et al. Ventral striatal plasticity and spatial memory. Proc. Natl. Acad. Sci. U. S. A. 107, 7945–50 (2010).

## SUPPLEMENTARY TABLES

### Supplementary Table 1. Detail information for figures and supplementary figures.

| Main Experiments | Pharmacologic reagent | Behavioral task | N | Figure |
|---|---|---|---|---|
| Retrograde tracing (FG) + fos activation | / | / | 8 | 1; S1 |
| | / | cMWM | 8 | |
| | / | sMWM | 9 | |
| Contralateral vSUB/VS disconnection | PBS | sMWM | 9 | 2a; S3a-c; S4c |
| | AP-5 | | 10 | |
| Ipsilateral vSUB/VS disconnection | PBS | sMWM | 11 | 2b; S3d-f; S4d |
| | AP-5 | | 10 | |
| Contralateral vSUB/VS disconnection | PBS | ODT | 15 | 2c; S5; S7a |
| | AP-5 | | 9 | |
| Contralateral vSUB/VS disconnection | PBS | cMWM | 8 | 2d; S6; S7b |
| | AP-5 | | 9 | |
| Unilateral vSUB inhibition + VS dendritic spine density | PBS (hemispheres) | cMWM | 5 | 3; S8 |
| | AP-5 (hemispheres) | | 5 | |
| | PBS (hemispheres) | sMWM | 7 | |
| | AP-5 (hemispheres) | | 5 | |
| Acute slice recording in vSUB-projecting VS neurons | vSUB DREADDs/VS CNO | / | 13 cells; 6 mice | 4a-d; S9a-d |
| | VS CNO | / | 7 cells; 5 mice | S9e-g |
| vSUB/VS DREADDs-mediated pathway inhibition | PBS | sMWM | 7 | 4e-g; S10;S11a-c; S14a |
| | CNO | | 13 | |
| vSUB/VS DREADDs-mediated pathway inhibition 120 min post-training | PBS | sMWM | 11 | 4h; S11d-g; S14b |
| | CNO | | 9 | |
| **Supplementary Experiments** | **Pharmacologic reagent** | **Behavioral task** | **N** | **Figure** |
| Retrograde tracing | FG | naive/cMWM/sMWM | 7 | S1 |
| Bilateral vSUB inhibition | PBS | sMWM | 11 | S2a-c; S4a |
| | AP-5 | | 8 | |
| Bilateral VS inhibition | PBS | sMWM | 13 | S2d-f; S4b |
| | AP-5 | | 13 | |
| vSUB/VS DREADDs-aspecific effects | PBS | EPM/OF/EO | 7 | S12 |
| | CNO | | 5 | |
| CNO aspecific effects controls | PBS | sMWM | 16 | S13a-c; S14c |
| | CNO | | 13 | |
| | PBS | Open field | 7 | S13d-i; S14d |
| | CNO | | 7 | |

## SUPPLEMENTARY FIGURES

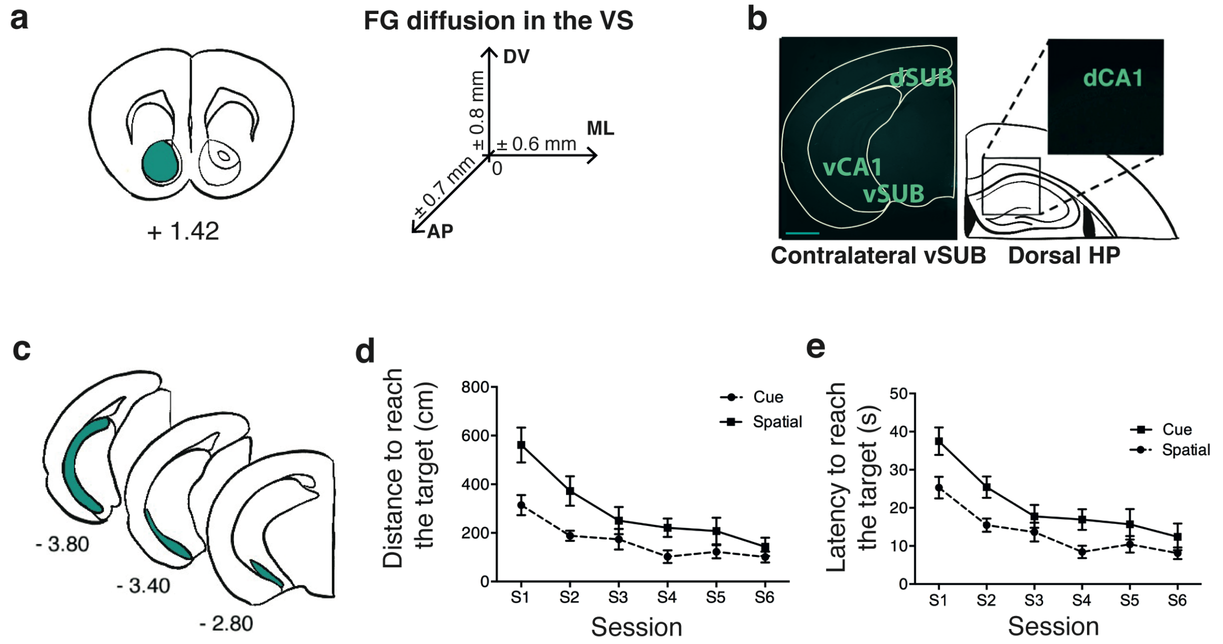


**Supplementary Figure 1. HPC and SUB labeling after unilateral VS Fluoro-Gold injections in cue and spatial trained mice.**

**a.** Schematic of FG labeling in the VS. FG labeled a sphere with a radius of approximately 0.7 mm from the injection site (AP= +1.42 mm from bregma) (n = 7). **b.** Microphotographs of a coronal section of the vSUB contralateral to the injected VS and of the dorsal HPC, ipsilateral to the injected VS, showing no retrograde tracing. Scale bar: 1 mm. **c.** Schematic representation of FG labeling along the antero-posterior axis of the hippocampal formation ipsilateral to the injected VS. Retrogradely labeled cells were detected starting from AP coordinate -2.80 mm, extending caudally to -3.80 mm from bregma. Rostrally, labeling was found mainly in the ventral CA1-CA3, extending more caudally to the whole SUB (n = 7). **d.** Path length expressed as mean distance (cm) ± SEM during spatial (n= 9) and cue (n= 8) training in the Morris water maze [two-way ANOVA of training $F_{1,15}$ =8.441, p = 0.0109; path-length $F_{5,75}$ = 18.913, p < 0.0001; training x path-length $F_{5,75}$ = 2.074, p = 0.0780; cue training ANOVA: $F_{5,35}$ = 8.976, p < 0.0001; spatial training ANOVA: $F_{5,40}$ = 11.997, p < 0.0001]. **e.** Mean latency to reach the target (s) ± SEM during spatial and cue training Morris water maze [two-way ANOVA of training $F_{1,15}$ =14.554, p = 0.0017; latency $F_{5,75}$ = 16.018, p < 0.0001; training x latency $F_{5,75}$ = 0.740, p = 0.5957; cue training ANOVA: $F_{5,35}$ =9.872, p < 0.0001; spatial training ANOVA: $F_{5,40}$ = 8.445, p < 0.0001].

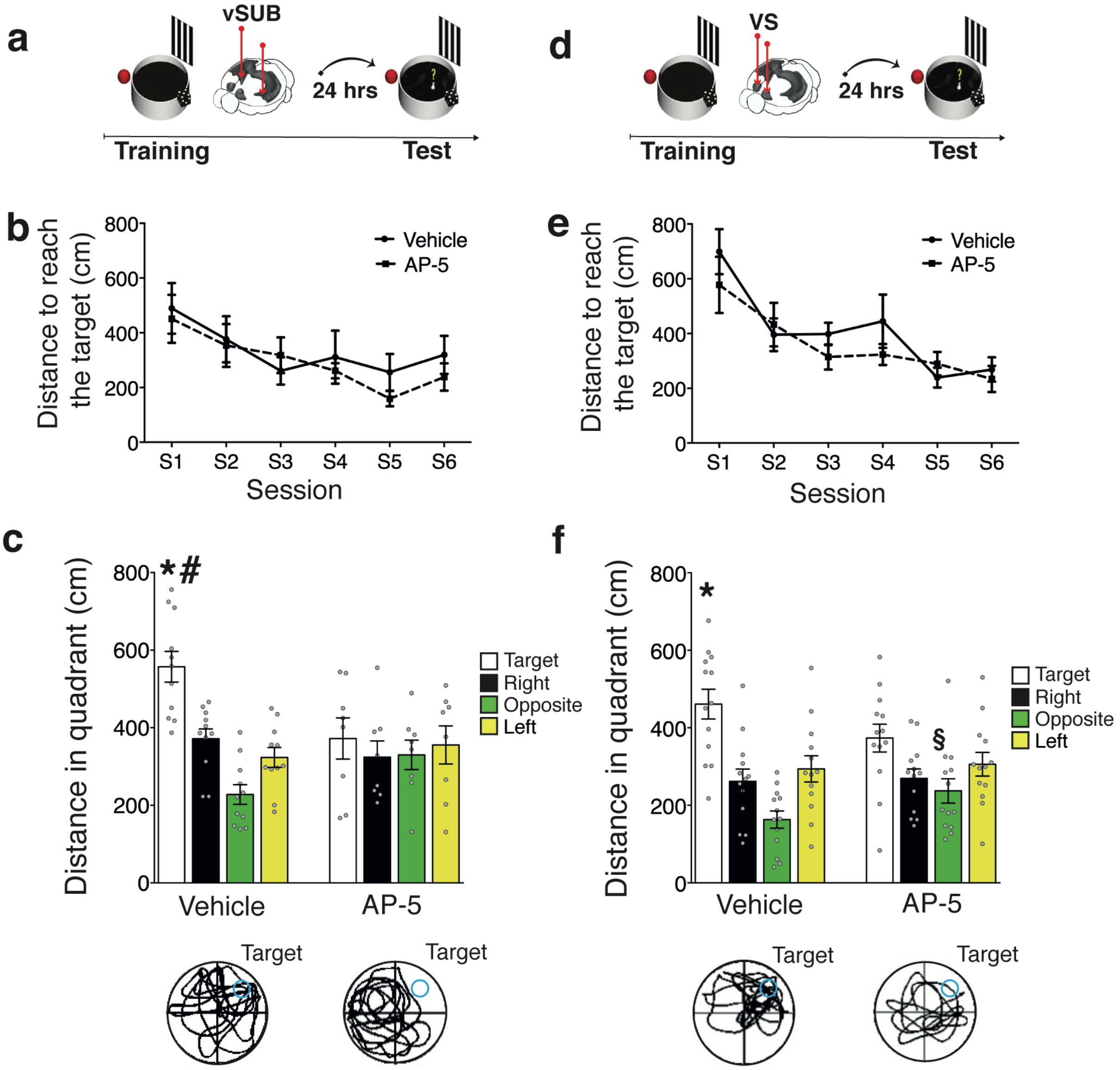
a
vSUB
24 hrs
Training
Test
d
VS
24 hrs
Training
Test
b
Distance to reach the target (cm)
Vehicle
AP-5
Session
e
Distance to reach the target (cm)
Vehicle
AP-5
Session
c
Distance in quadrant (cm)
Target
Right
Opposite
Left
Vehicle
AP-5
f
Distance in quadrant (cm)
Target
Right
Opposite
Left
Vehicle
AP-5

**Supplementary Figure 2. Bilateral inhibition of vSUB and VS after spatial training in the MWM.** **a.** Schematic representation of the experimental design. MWM images modified from (PNAS 107, 7945-50 (2010); Image credit for brains schematics: Allen Institute. **b.** Path length expressed as mean distance (cm) ± SEM during spatial training in the Morris water maze before bilateral vSUB vehicle (n = 11) or AP-5 (n = 8) administrations (two-way ANOVA effect of sessions $F_{5,85}$ =5.921; p < 0.0001; treatment $F_{1,17}$ = 0.089; p = 0.7691; session x treatment $F_{5,85}$ = 0.498; p = 0.7766). **c.** Distance travelled in the four quadrants 24h after bilateral administrations of vehicle or AP-5 in the vSUB [two-way ANOVA of quadrants preference $F_{3,51}$ = 7.360; p = 0.0003; treatment $F_{1,17}$ = 2.190; p = 0.1572; quadrants preference x treatment $F_{3,51}$ = 4.632; p = 0.0061]. Post-hoc analysis (Tukey HSD) revealed that PBS, but not AP-5 injected mice were able to correctly locate the platform position. In the bottom panels are shown representative probe-trial paths. **d.** Schematic representation of the experimental design. **e.** Path length expressed as mean distance (cm) ± SEM during spatial training in the Morris water maze before bilateral VS vehicle (n = 13) or AP-5 (n = 13) administrations [two-way ANOVA of sessions $F_{5,120}$ = 9.885; p < 0.0001; treatment $F_{1,24}$ = 1.314; p = 0.2630; session x treatment $F_{5,120}$ = 0.734; p = 0.5990]. **f.** Distance travelled in the four quadrants 24h after bilateral administrations of vehicle or AP-5 in the VS [two-way ANOVA of quadrants preference $F_{3,72}$ = 14.767; p < 0.0001; treatment $F_{1,24}$ = 0.008; p = 0.9313; quadrants preference x treatment $F_{3,72}$ = 1.981; p = 0.1244; vehicle group: one-way ANOVA of quadrants preference $F_{3,36}$ = 12.587; p < 0.0001; AP-5 group: one-way ANOVA of quadrants preference $F_{3,36}$ = 3.348; p = 0.0296]. Post-hoc analysis (Tukey HSD) revealed that PBS, but not AP-5 injected mice were able to correctly locate the platform position. In the bottom panels are shown representative probe-trial paths. * p < 0.05 target *vs* right, opposite, left (within group); # p < 0.05 target *vs* target (between groups); § p < 0.05 *vs* target (within group); Tukey HSD.

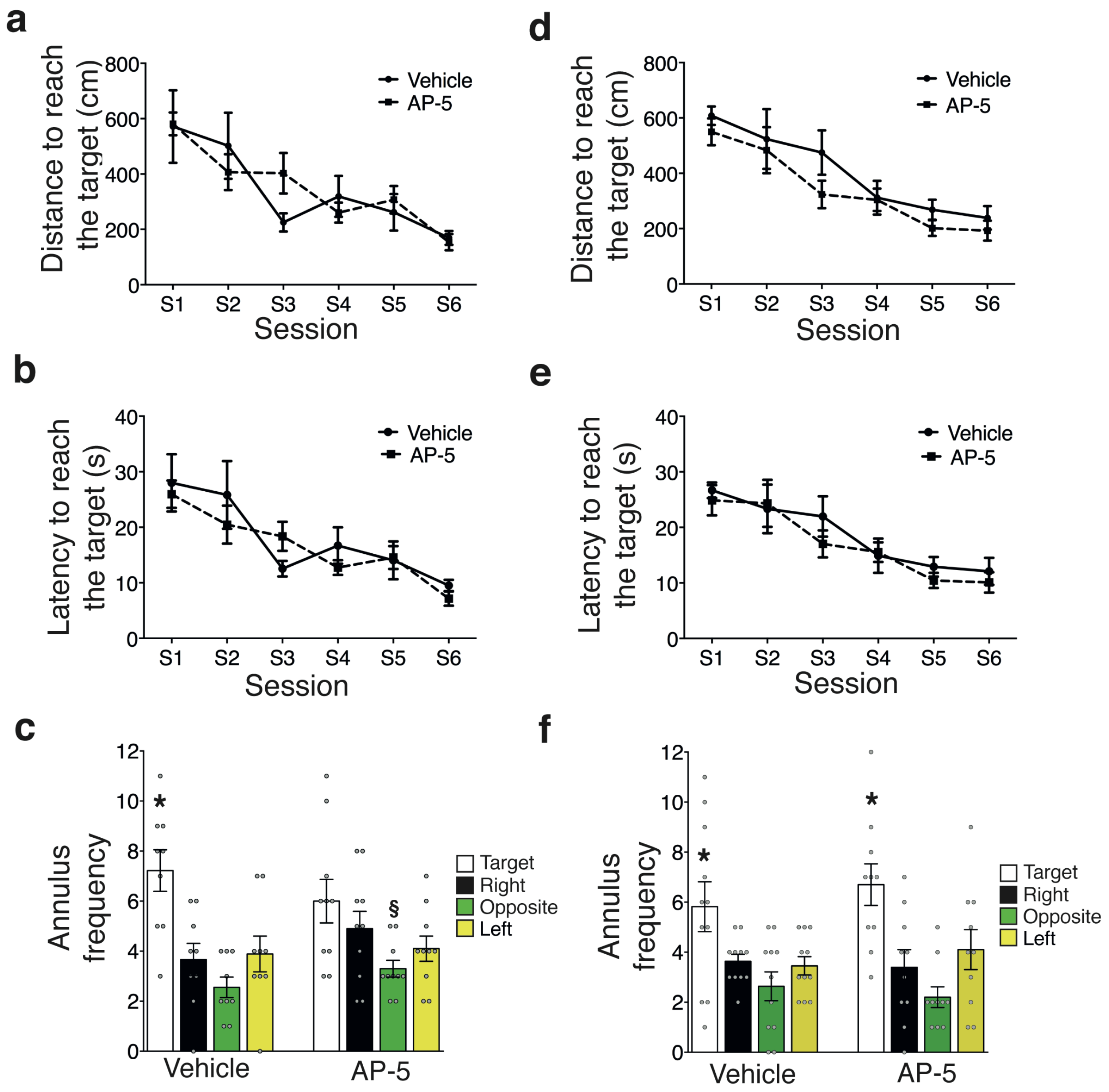
a
Distance to reach the target (cm)
Vehicle
AP-5
Session
S1
S2
S3
S4
S5
S6
b
Latency to reach the target (s)
Vehicle
AP-5
Session
c
Annulus frequency
Target
Right
Opposite
Left
Vehicle
AP-5
d
Distance to reach the target (cm)
Vehicle
AP-5
Session
e
Latency to reach the target (s)
Vehicle
AP-5
Session
f
Annulus frequency
Target
Right
Opposite
Left
Vehicle
AP-5

**Supplementary Figure 3. Training performance for contralateral and ipsilateral vSUB-VS disconnected groups. a.** Mean of path length (cm) ± SEM during spatial Morris water maze training sessions before unilateral vSUB and contralateral VS vehicle or AP-5 administrations [two-way ANOVA of sessions $F_{5,85} = 11.548$; $p < 0.0001$; treatment $F_{1,17} = 0.035$; $p = 0.8544$; session x treatment $F_{5,85} = 1.240$; $p = 0.2979$]. **b.** Mean of latency (s) ± SEM during sMWM training sessions before unilateral vSUB and contralateral VS vehicle or AP-5 administrations [two-way ANOVA of sessions $F_{5,85} = 11.684$; $p < 0.0001$; treatment $F_{1,17} = 0.243$; $p = 0.6282$; session x treatment $F_{5,85} = 1.018$; $p = 0.4122$]. **c.** Mean annulus frequency ± SEM on probe trial after unilateral vSUB and contralateral VS vehicle or AP-5 administrations [two-way ANOVA of annuli $F_{3,51} = 10.454$; $p < 0.0001$; treatment $F_{1,17} = 0.376$; $p = 0.5478$; annuli x treatment $F_{3,51} = 1.223$; $p = 0.3108$; one way ANOVA of vehicle: $F_{3,24}= 8.1238$; $p= 0.0007$; of AP-5: $F_{3,27}= 3.124$; $p= 0.0423$]. **d.** Mean of path length (cm) ± SEM during sMWM training sessions before unilateral vSUB and ipsilateral VS vehicle or AP-5 administrations [two-way ANOVA of sessions $F_{5,95} = 14.367$; $p < 0.0001$; treatment $F_{1,19} = 1.814$; $p = 0.1938$; session x treatment $F_{5,95} = 0.388$; $p = 0.8556$]. **e.** Mean of latency (s) ± SEM during spatial Morris water maze training sessions before unilateral vSUB and ipsilateral VS vehicle or AP-5 administrations [two-way ANOVA of sessions $F_{5,95} = 11.824$; $p < 0.0001$; treatment $F_{1,19} = 0.522$; $p = 0.4786$; session x treatment $F_{5,95} = 0.366$; $p = 0.8705$]. **f.** Mean annulus frequency ± SEM on probe trial after unilateral vSUB and ipsilateral VS vehicle or AP-5 administrations [two-way ANOVA of annuli $F_{3,57} = 11.167$; $p < 0.0001$; treatment $F_{1,19} = 0.270$; $p = 0.6093$; annuli x treatment $F_{3,57} = 0.445$; $p = 0.7216$; one way ANOVA of vehicle: $F_{3,30}= 4.987$; $p= 0.0063$; of AP-5: $F_{3,27}= 6.151$; $p= 0.0025$]. * $p < 0.05$ target *vs* right, opposite, left (within group); § $p < 0.05$ *vs* target (within group), Tukey HSD.

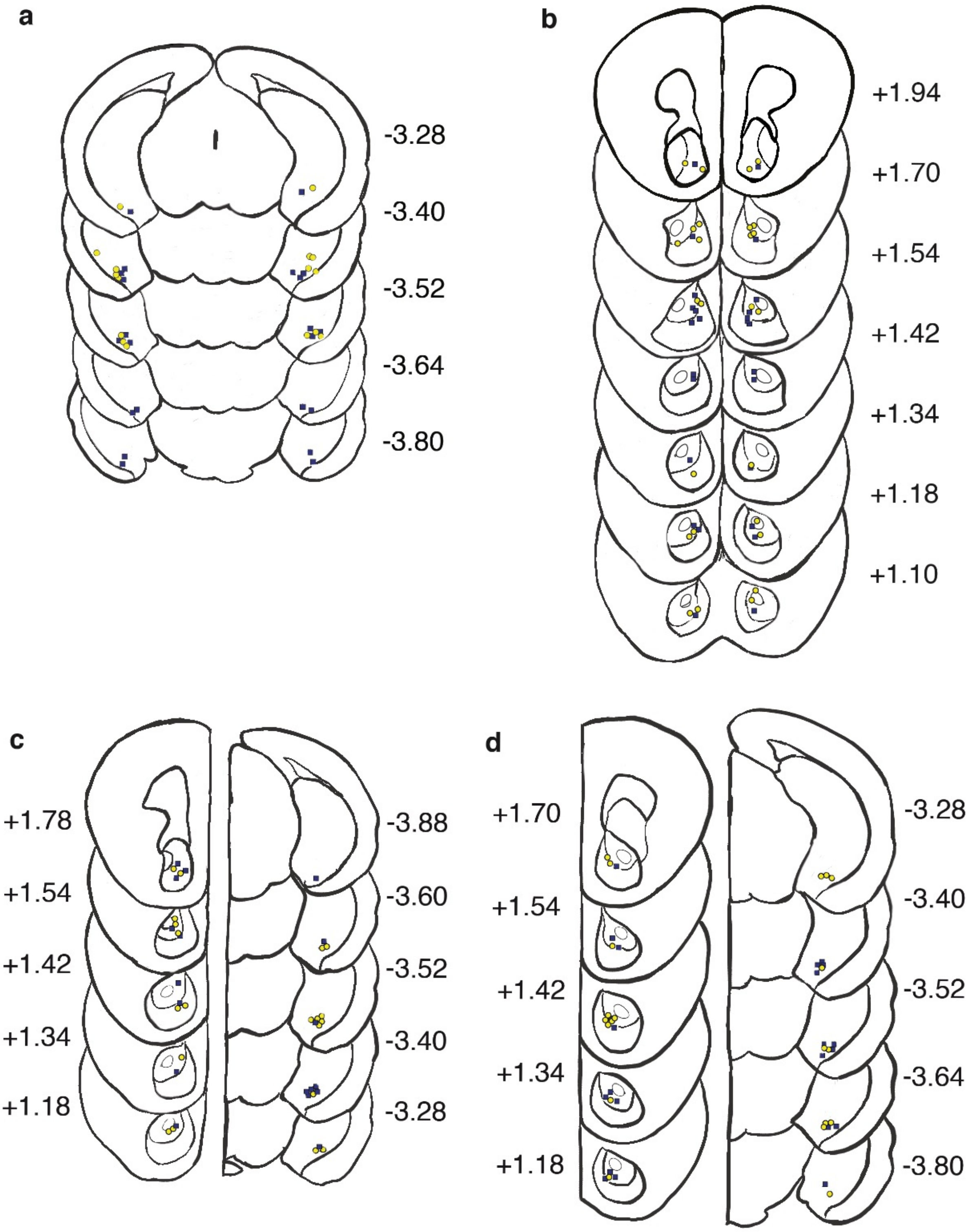
a
-3.28
-3.40
-3.52
-3.64
-3.80
b
+1.94
+1.70
+1.54
+1.42
+1.34
+1.18
+1.10
c
+1.78
+1.54
+1.42
+1.34
+1.18
-3.88
-3.60
-3.52
-3.40
-3.28
d
+1.70
+1.54
+1.42
+1.34
+1.18
-3.28
-3.40
-3.52
-3.64
-3.80

**Supplementary Figure 4. Schematic representation of injection sites for the pharmacology experiments in the MWM.** Each symbol represents the site of injection for one animal. **a.** Post-training vSUB vehicle (blue squares, n = 11) and AP-5 (yellow circles, n = 8) injection sites. **b.** Post-training VS vehicle (blue squares, n = 13) and AP-5 (yellow circles, n = 13) injection sites. **c.** Post-training vSUB and contralateral VS injection sites for the vehicle (blue squares, n = 9) and the AP-5 (yellow circles, n = 10) groups. **d.** Post-training vSUB and ipsilateral VS injection sites for the vehicle (blue squares, n = 11) and the AP-5 groups (yellow circles, n = 10). Coordinates are expressed as mm from bregma.

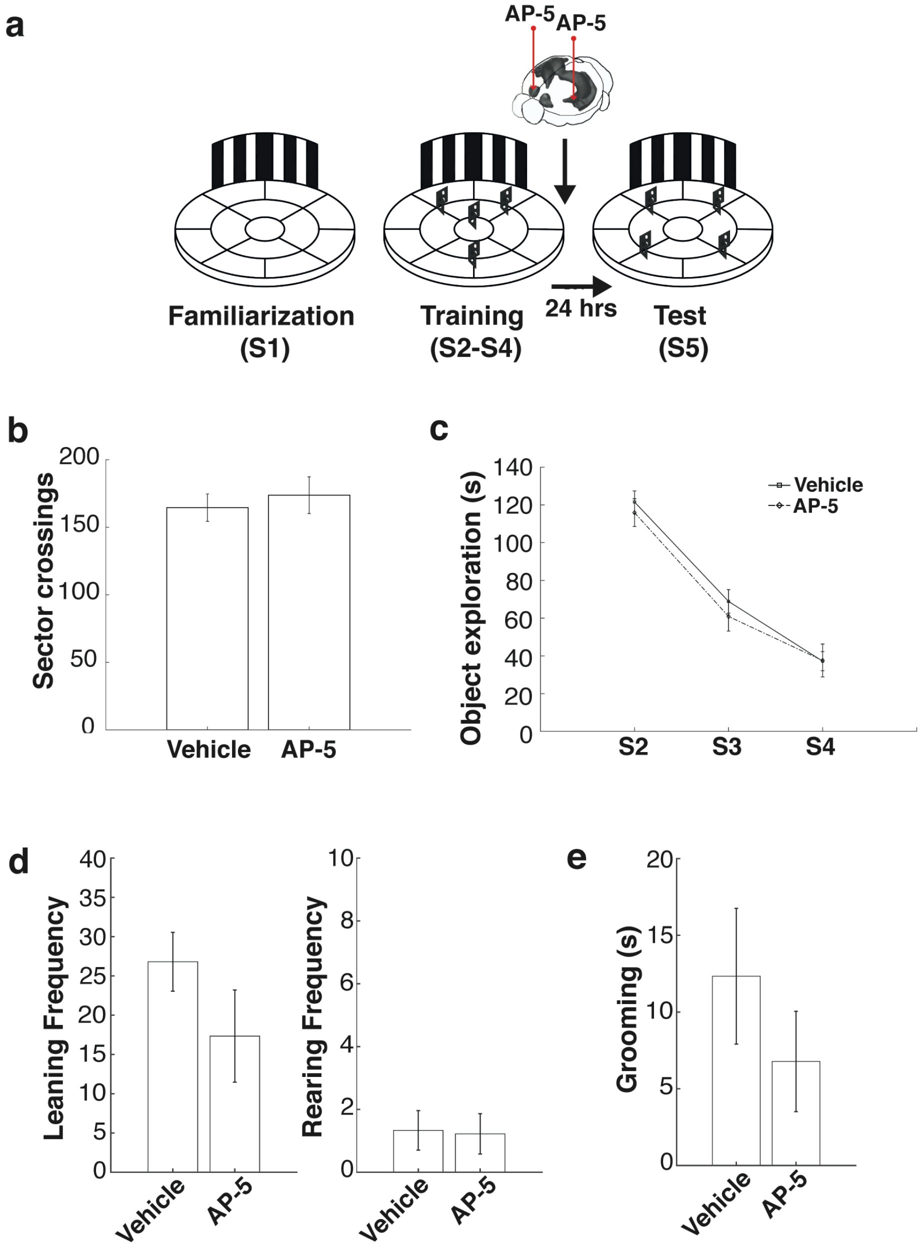


**Supplementary Figure 5. vSUB-VS contralateral disconnection in the object displacement task (ODT). a.** Schematic representation of the experimental design. S1 and S2 to S4 were interspaced with a 2 min intertrial interval (ITI); probe test at S5 was performed 24h after training. Image credit for brains schematics: Allen Institute. **b.** Locomotor activity of mice in the S1 showing no difference between groups before treatment [$F_{1,22} = 0.300$; $p = 0.5896$]. **c.** Object exploration during the training phase showing a progressive decrease of exploration across sessions before treatment [two-way ANOVA effect of sessions $F_{2,44} = 156.161$; $p < 0.0001$; treatment $F_{1,22} = 0.305$; $p = 0.5863$; session x treatment $F_{2,44} = 0.422$; $p = 0.6586$]. **d.** Exploratory behavior measured as leaning (on the left) and rearing (on the right) frequency during the S5 for vehicle and AP-5 injected mice. Data show no differences between groups indicating no side-effects of the post-training injection procedure on mice exploration [leaning $F_{1,22} = 2.042$; $p = 0.1670$; rearing $F_{1,22} = 0.014$; $p = 0.9084$]. **e.** Grooming in S5; no difference was found between vehicle and AP-5 injected mice [$F_{1,22} = 0.782$; $p = 0.3862$].

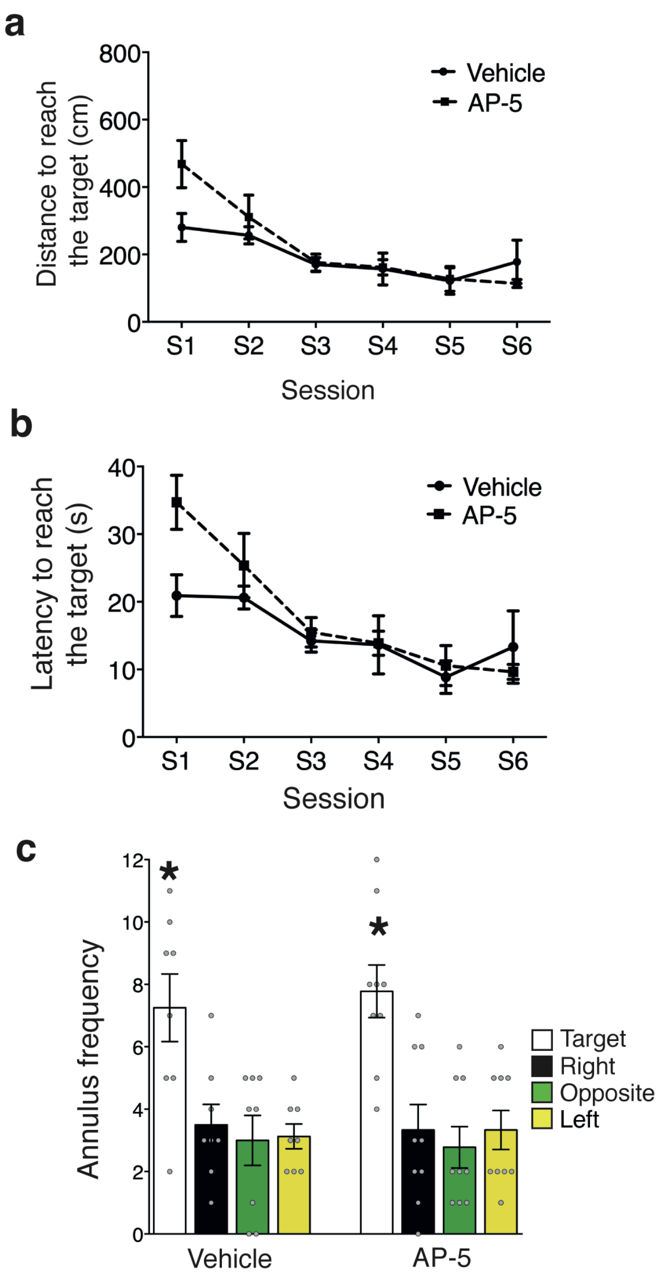


**Supplementary Figure 6. Training performance for the contralateral vSUB-VS disconnected group trained in the cMWM.a.** Mean of path length (cm) ± SEM during cue Morris water maze training sessions before unilateral vSUB and contralateral VS vehicle or AP-5 administrations [two-way ANOVA of sessions $F_{5,75}$ =11.284; p < 0.0001; treatment $F_{1,15}$ = 1.101; p = 0.3106; session x treatment $F_{5,75}$= 2.140; p = 0.0698]. **b.** Mean of latency (s) ± SEM during cue Morris water maze training sessions before unilateral vSUB and contralateral VS vehicle or AP-5 administrations [two-way ANOVA of sessions $F_{5,75}$ =11.192; p < 0.0001; treatment $F_{1,15}$ = 1.581; p = 0.2278; session x treatment $F_{5,75}$= 2.040; p = 0.0827]. **c.** Mean annulus frequency ± SEM on probe trial after unilateral vSUB and contralateral VS vehicle or AP-5 administrations [two-way ANOVA of annuli $F_{3,45}$ = 15.607; p < 0.0001; treatment $F_{1,15}$ = 0.031; p = 0.8624; annuli x treatment $F_{3,45}$ = 0.101; p = 0.9590; one way ANOVA of vehicle: $F_{3,21}$= 6.504; p= 0.0028; of AP-5: $F_{3,24}$= 9.361; p= 0.0003]. * p < 0.05 target *vs* right, opposite, left (within group), Tukey HSD.

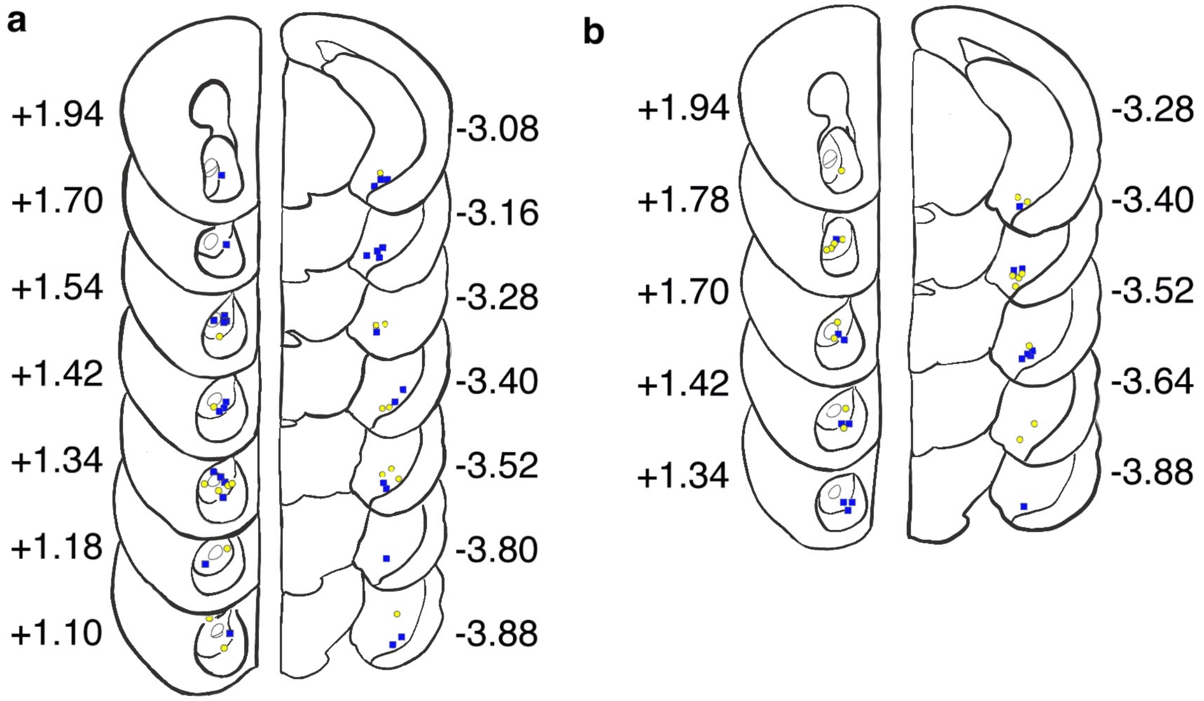


**Supplementary Figure 7. ODT and cMWM schematic representation of injection sites for pharmacology experiments.** Each symbol represents the site of injection for one animal. **a.** Schematic representation of the post-training vSUB and contralateral VS injection sites in the ODT experiment for the vehicle (blue squares, n = 15) and the AP-5 (yellow circles, n = 9) injected groups. **b.** Schematic representation of the post-training vSUB and contralateral VS injection sites in the cMWM functional disconnection experiment for the vehicle (blue squares, n = 8) and AP-5 (yellow circles, n = 9) injected groups. Coordinates are expressed as mm from bregma.

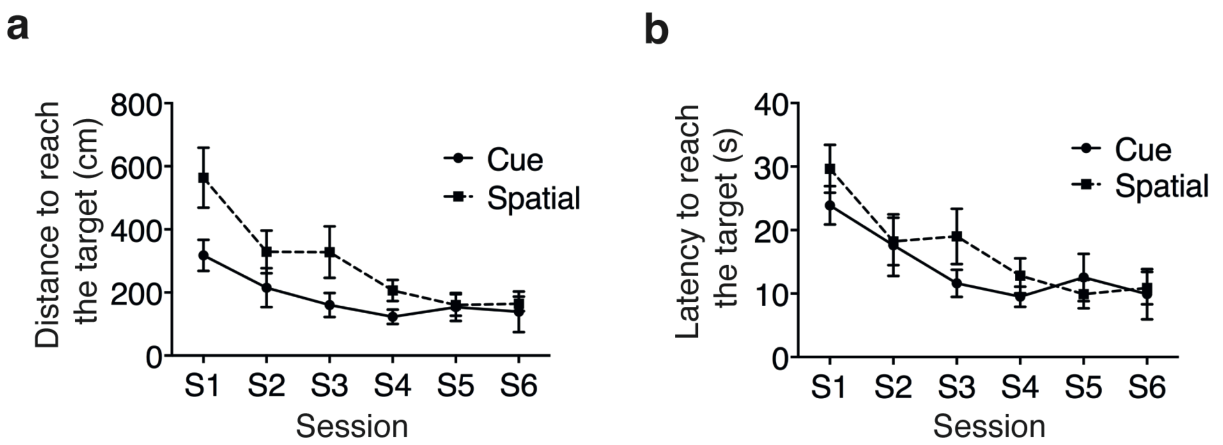


**Supplementary Figure 8. Training performance of mice trained in the cMWM and sMWM for spine density experiment. a.** Mean of path length (cm) ± SEM during cue and spatial MWM training sessions [two-way ANOVA of sessions $F_{5,45} = 7.147$; $p < 0.0001$; training $F_{1,9} = 13.179$; $p = 0.0055$; session x training $F_{5,45} = 1.173$; $p = 0.3374$]. **b.** Mean of latency (s) ± SEM during cue and spatial Morris water maze training sessions [two-way ANOVA of sessions $F_{5,45} = 6.779$; $p < 0.0001$; training $F_{1,9} = 1.865$; p = 0.20 5; session x training $F_{5,45} = 0.580$; $p = 0.7149$].

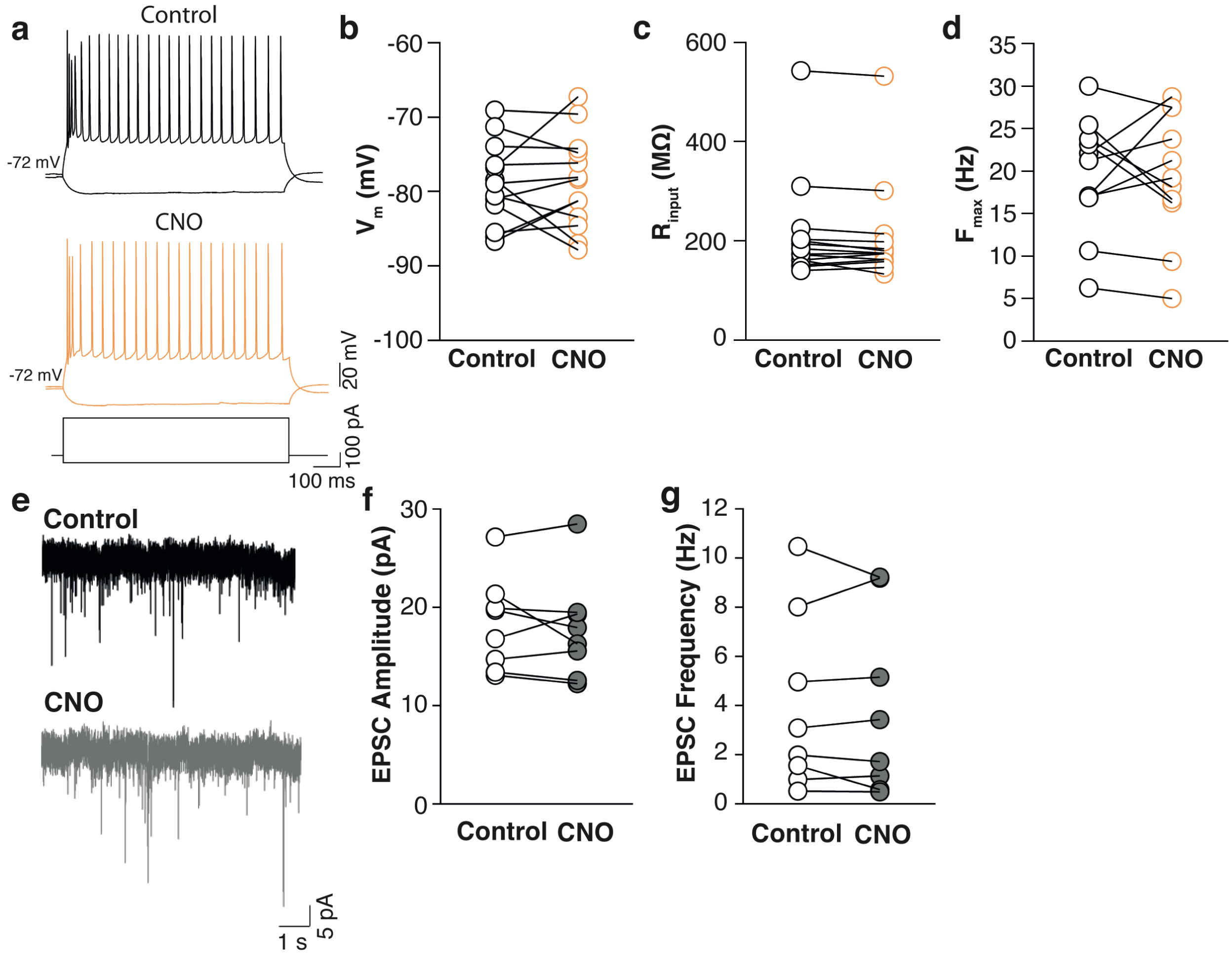


**Supplementary Figure 9. CNO does not change intrinsic membrane properties of MSNs. a.** Representative traces showing voltage responses of MSNs to hyperpolarizing and depolarizing current steps in control (black) and during CNO 10 µM (orange) bath-application recorded 5-8 weeks after AAV-Syn::Venus-2A-HA-hM4D$_i$ injection in the vSUB (n=6 mice). **b** Scatter plot graph summarizing Resting membrane potentials ($V_m$) in control (black) and during CNO 10 µM (orange) bath-application [n = 13 cells; p = 0.89; Wilcoxon matched-pairs signed rank test]. **c** Scatter plot graph summarizing input resistance ($R_{input}$) in control (black) and during CNO 10 µM (orange) bath-application [n = 13 cells; p = 0.23; Wilcoxon matched-pairs signed rank test]. **d** Scatter plot graph summarizing the maximal firing frequency in response to depolarizing current steps (300 pA) in control (black) and during CNO 10 µM (orange) bath-application [n = 11 cells; p = 0.98; Wilcoxon matched-pairs signed rank test]. **e.** Representative traces from VS MSNs of naïve mice which did not receive viral injection (n = 5 mice) showing sEPSCs in control conditions (black) and during CNO application (gray). **f.** In slice from naïve mice CNO (10 µM) had no effect on sEPSCs amplitude [n = 8 cells; $t_7$ = 0.66, p = 0.53, paired t-test] or **g.** frequency [$t_7$ = 0.32, p = 0.76, paired t-test].

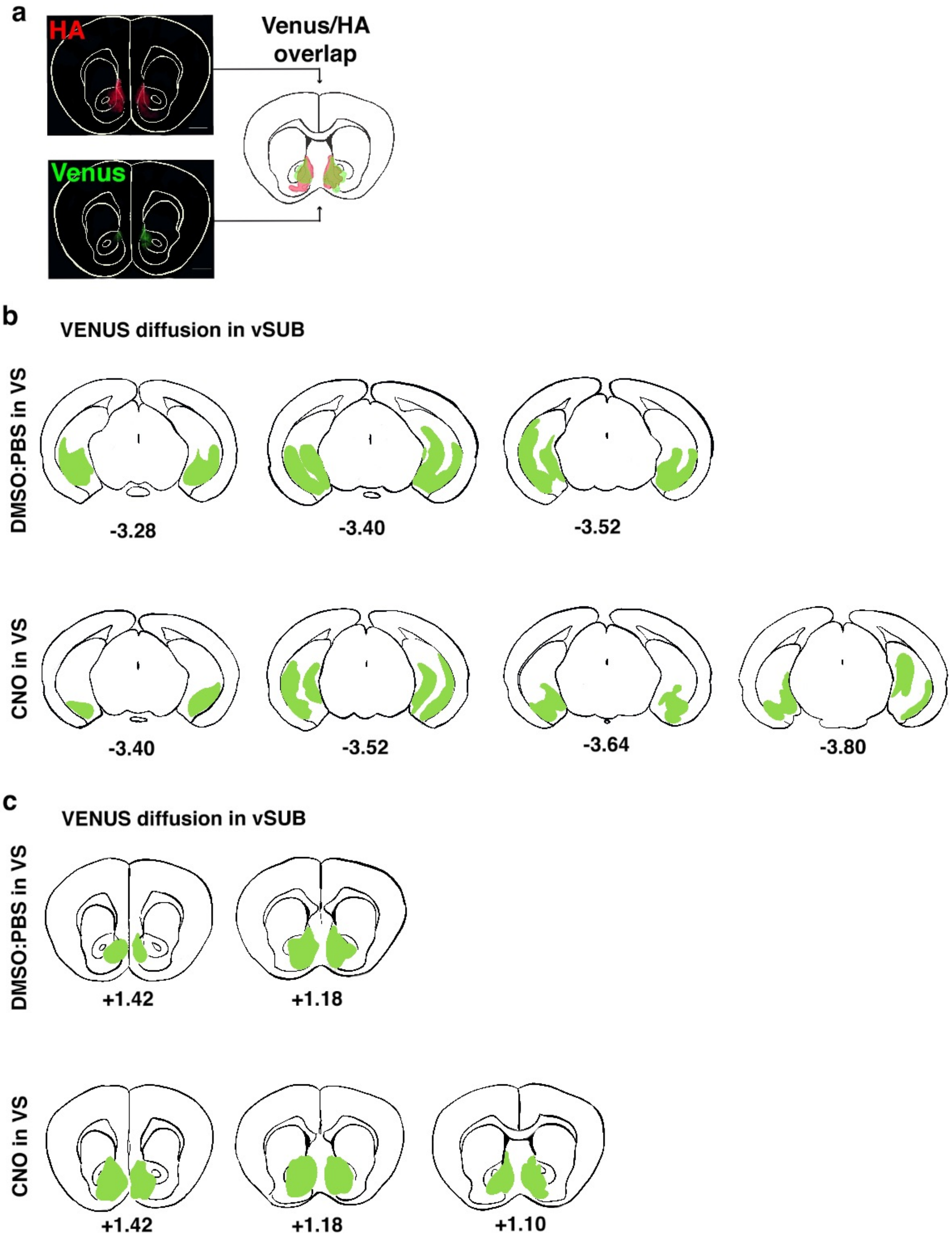
a
HA
Venus
Venus/HA overlap
b
VENUS diffusion in vSUB
DMSO:PBS in VS
-3.28
-3.40
-3.52
CNO in VS
-3.40
-3.52
-3.64
-3.80
c
VENUS diffusion in vSUB
DMSO:PBS in VS
+1.42
+1.18
CNO in VS
+1.42
+1.18
+1.10

**Supplementary Figure 10. AAV-Syn::Venus-2A-HA-hM4D$_i$ virus diffusion in vSUB and VS. a.** Representative microphotographs for HA (red) and Venus protein (green) expression in the VS after AAV-Syn::Venus-2A-HA-hM4D$_i$ injection in the vSUB. Mean overlap of the two signals in the VS was of 80 ± 5% (n slices = 12). **b-c.** Schematic representation of Venus protein expression in vSUB and in the VS respectively. Venus maximum extension is represented in green for each single mouse. Venus in the VS labeled a sphere of approximately 0.757 ± 0.093 mm$^2$ in vehicle injected mice and of 0.929 ± 0.085 mm$^2$ in CNO injected mice.

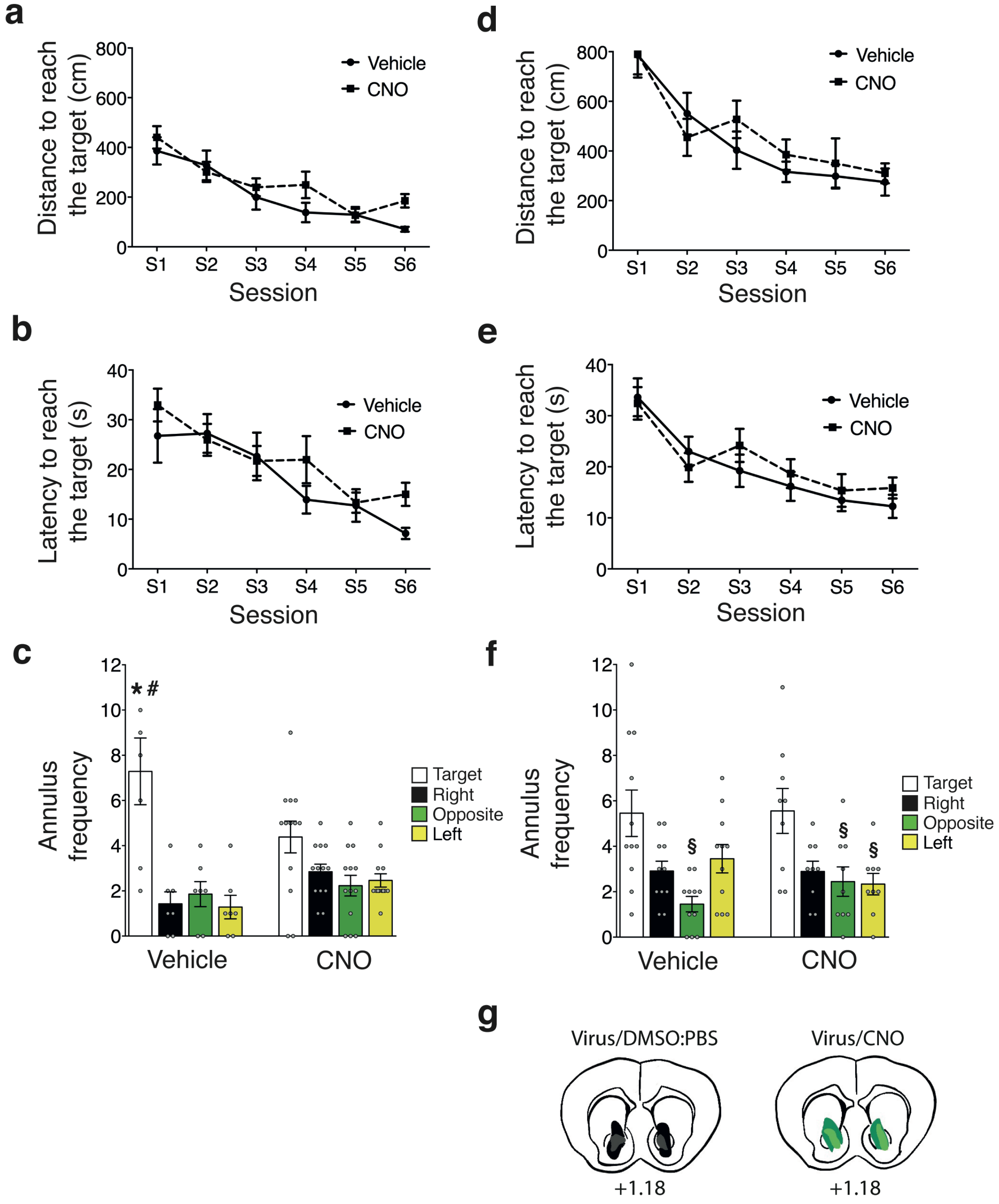
a
Distance to reach the target (cm)
Session
Vehicle
CNO
b
Latency to reach the target (s)
Session
Vehicle
CNO
c
Annulus frequency
*#
Target
Right
Opposite
Left
Vehicle
CNO
d
Distance to reach the target (cm)
Session
Vehicle
CNO
e
Latency to reach the target (s)
Session
Vehicle
CNO
f
Annulus frequency
§
Target
Right
Opposite
Left
Vehicle
CNO
g
Virus/DMSO:PBS
Virus/CNO
+1.18
+1.18

**Supplementary Figure 11. Analysis of performance in the chemogenetic experiments in the sMWM. a.** Mean of path length (cm) ± SEM during sMWM training [two-way ANOVA of sessions $F_{5,90}$ = 15.226; p < 0.0001; treatment $F_{1,18}$ = 1.998; p = 0.1746; session x treatment $F_{5,90}$ = 1.004; p = 0.4199]. **b.** Mean of latency (s) ± SEM during sMWM training sessions [two-way ANOVA of sessions $F_{5,90}$ = 12.024; p < 0.0001; treatment $F_{1,18}$ = 2.641; p = 0.1215; session x treatment $F_{5,90}$ = 0.717; p = 0.6126]. **c.** Mean annulus frequency ± SEM on probe trial after post-training VS vehicle or CNO administrations [two-way ANOVA of annuli $F_{3,54}$ = 16.414; p < 0.0001; treatment $F_{1,18}$ = 0.002; p = 0.9665; annuli x treatment $F_{3,54}$ = 4.472; p = 0.0071]. * p < 0.05 target *vs* others (within group); # p < 0.05 target *vs* target (between groups); Tukey HSD. **d.** Mean of path length (cm) ± SEM during sMWM training sessions before 120 min post-training vehicle or CNO administrations in the VS [two-way ANOVA of sessions $F_{5,90}$ = 17.0220; p < 0.0001; treatment $F_{1,18}$ = 0.3695; p = 0.5508; session x treatment $F_{5,90}$ = 0.8005; p = 0.5521]. **e.** Mean of latency (s) ± SEM during sMWM training sessions before 120 min post-training vehicle or CNO administrations in the VS [two-way ANOVA of sessions $F_{5,90}$ = 13.0922; p < 0.0001; treatment $F_{1,18}$ = 0.6103; p = 0.4448; session x treatment $F_{5,90}$ = 0.5723; p = 0.7210] **f.** Mean annulus frequency ± SEM on probe trial after 120 min post-training vehicle or CNO administrations in the VS [two-way ANOVA of annuli $F_{3,54}$ = 8.6617; p = 0.000089; treatment $F_{1,18}$ = 0.0017; p = 0.9678; annuli x treatment $F_{3,54}$ = 0.694; p = 0.5597; vehicle group: one-way ANOVA of quadrants frequency $F_{3,30}$ = 5.4972; p = 0.0039; CNO group: one-way ANOVA of quadrants frequency $F_{3,24}$ = 4.0215; p = 0.01884]. § p < 0.05 *vs* target.; Tukey HSD **g.** Schematic representation of max (dark) and min (light) Venus protein expression in the VS for vehicle (black) and CNO (green) injected mice.

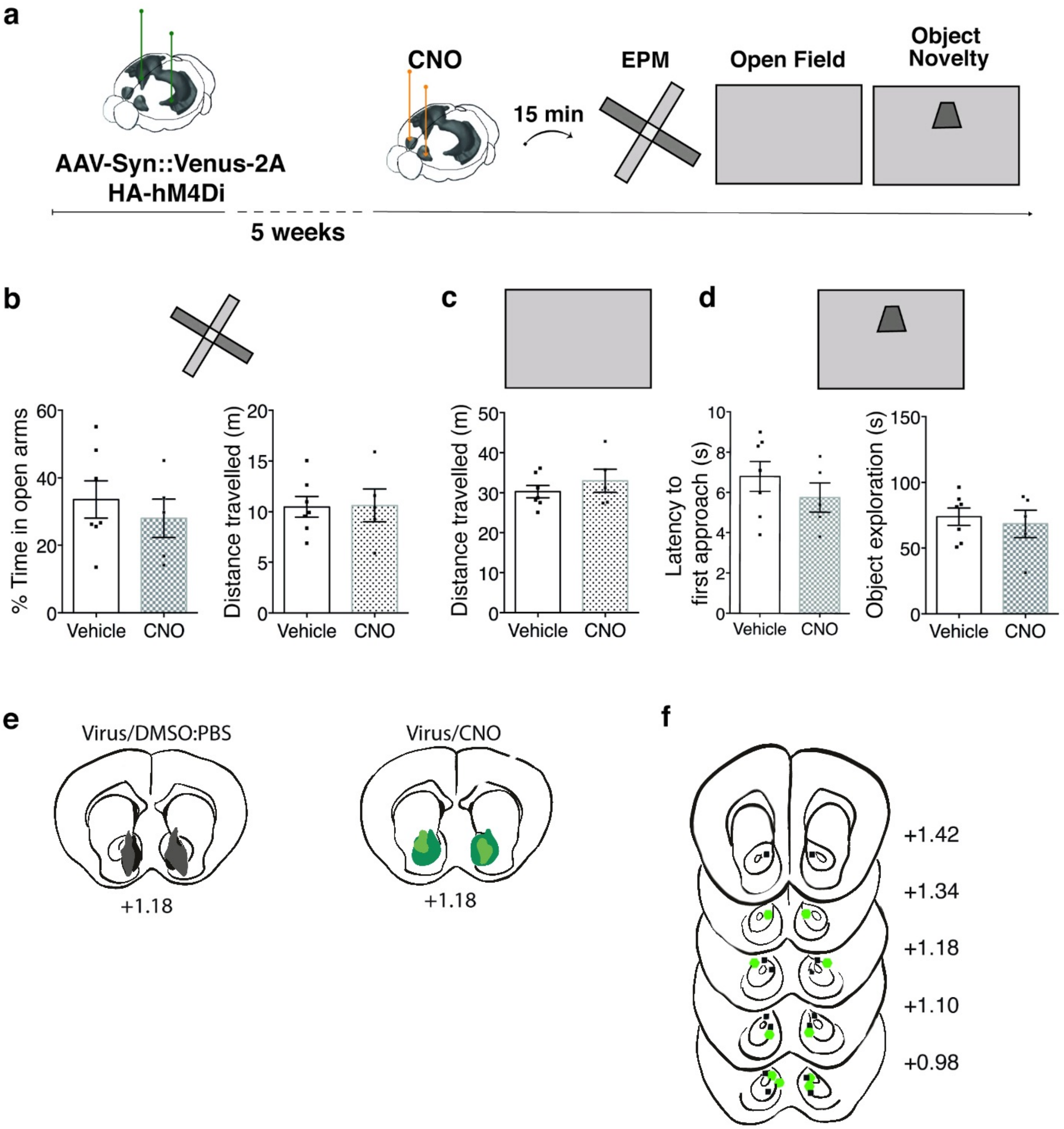

a
AAV-Syn::Venus-2A
HA-hM4Di
5 weeks
CNO
15 min
EPM
Open Field
Object
Novelty
b
% Time in open arms
Distance travelled (m)
Vehicle
CNO
c
Distance travelled (m)
d
Latency to
first approach (s)
Object exploration (s)
e
Virus/DMSO:PBS
Virus/CNO
+1.18
f
+1.42
+1.34
+1.18
+1.10
+0.98

**Supplementary Figure 12. Effects of vSUB-VS DREADDs inhibition on anxiety, exploratory and motivational levels. a.** Schematic of the experimental design. AAV-Syn::Venus-2A HA-hM4Di virus was bilaterally injected in the vSUB. Five weeks after mice were bilaterally administered with saline (n= 7) or CNO 10mM (n= 5) in the VS and submitted to the following paradigms: elevated plus maze (EPM), open field and object novelty. Image credit for brains schematics: Allen Institute**. b.** Percentage of time spent in the open arms (left panel) ($t_{10}$ = 0.6868; p = 0.5078; unpaired t-test) and total distance travelled (right panel) ($t_{10}$ = 0.0723; p = 0.9438; unpaired t-test) that did not differ between groups, demonstrating no significant effects of vSUB/VS pathway inhibition on anxiety levels. **c.** CNO administrations in the VS did not affect distance travelled in the open field compared to control mice ($t_{10}$ = 0.8929; p = 0.3929; unpaired t-test), indicating no significant effects on exploratory behavior and locomotion. **d.** Intra VS administrations of CNO did not affect latency to approach the novel object ($t_{10}$= 0.9674; p= 0.3562; unpaired t-test) and the time spent exploring the object ($t_{10}$ = 0.4587; p = 0.6562; unpaired t-test) compared to control mice. **e.** Schematic representation of max (dark) and min (light) Venus protein expression in the VS for vehicle (black) and CNO (green) injected mice. **f.** Illustration of the most ventral tip of the injector placement in the VS of vehicle (black squares) and CNO (green hexagons) injected mice.

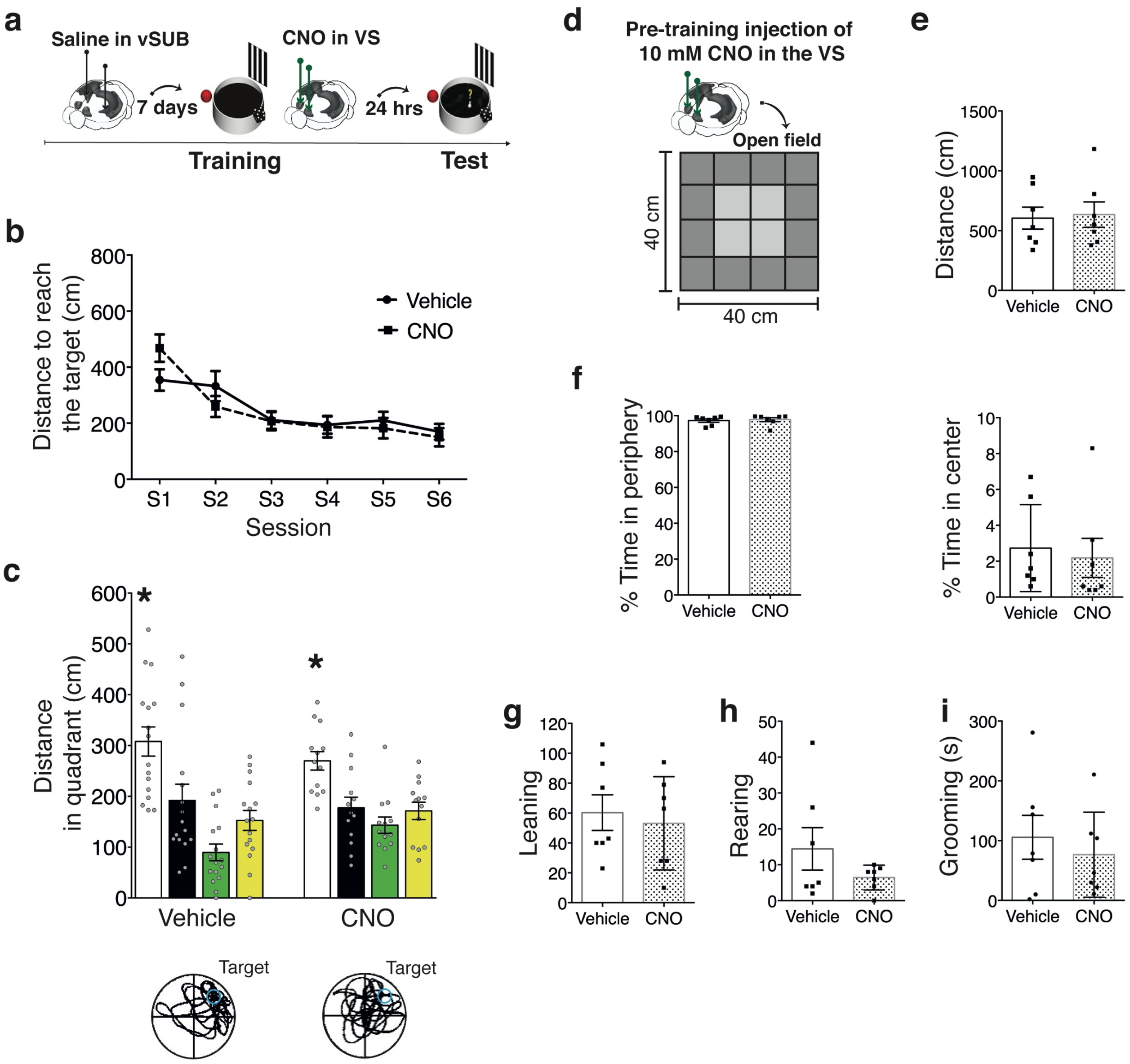

a
Saline in vSUB
7 days
CNO in VS
24 hrs
Training
Test
b
Distance to reach the target (cm)
Vehicle
CNO
Session
S1 S2 S3 S4 S5 S6
c
Distance in quadrant (cm)
Vehicle
CNO
Target
d
Pre-training injection of 10 mM CNO in the VS
Open field
40 cm
40 cm
e
Distance (cm)
f
% Time in periphery
% Time in center
g
Leaning
h
Rearing
i
Grooming (s)

**Supplementary Figure 13. Evaluation of non specific effects of 10 mM CNO injection in the VS. a.** Schematic of the experimental design. Mice received saline injection in the vSUB and post-training vehicle or CNO (10mM) focal administrations in the VS. . MWM images modified from (PNAS 107, 7945-50 (2010); Image credit for brains schematics: Allen Institute**. b.** Mean of path length (cm) ± SEM during sMWM training sessions before VS vehicle or CNO administrations [two-way ANOVA effect for sessions $F_{5,135} = 13.739$; $p < 0.0001$; treatment $F_{1,27} = 0.013$; $p = 0.9101$; session x treatment $F_{5,135} = 1.508$; $p = 0.1914$]. **c.** Distance travelled in the four quadrants 24h after bilateral administrations of vehicle (n = 16) or CNO (n = 13) in the VS [two-way ANOVA of quadrants preference $F_{3,81} = 18.838$; $p < 0.0001$; treatment $F_{1,27} = 1.129$; $p = 0.7219$; quadrants preference x treatment $F_{3,81} = 1.407$; $p = 0.2469$]. In the bottom panels are shown representative probe-trial path for the two experimental groups. **d.** Schematic of the experimental design. Mice were focally injected with vehicle or CNO (10mM) in the VS 15 min before being placed in the open field for 1 hr. ANOVA did not reveal significant differences for **e.** locomotor activity [one-way ANOVA: $F_{1,12} = 0.103$; $p = 0.7542$]; **f.** % time spent in the periphery and in the central quadrants [two-way ANOVA of quadrants preference $F_{1,12} = 4461.647$; $p < 0.0001$; treatment $F_{1,12} = 1.000$; $p = 0.3370$; quadrants x treatment $F_{1,12} = 0.149$; $p = 0.149$]; **g.** leaning [one-way ANOVA: $F_{1,8} = 0.0301$; $p = 0.8666$]; **h.** rearing frequency [one-way ANOVA: $F_{1,8} = 0.6791$; $p = 0.4338$], or **i.** grooming time [one-way ANOVA: $F_{1,8} = 0.0512$; $p = 0.8267$].

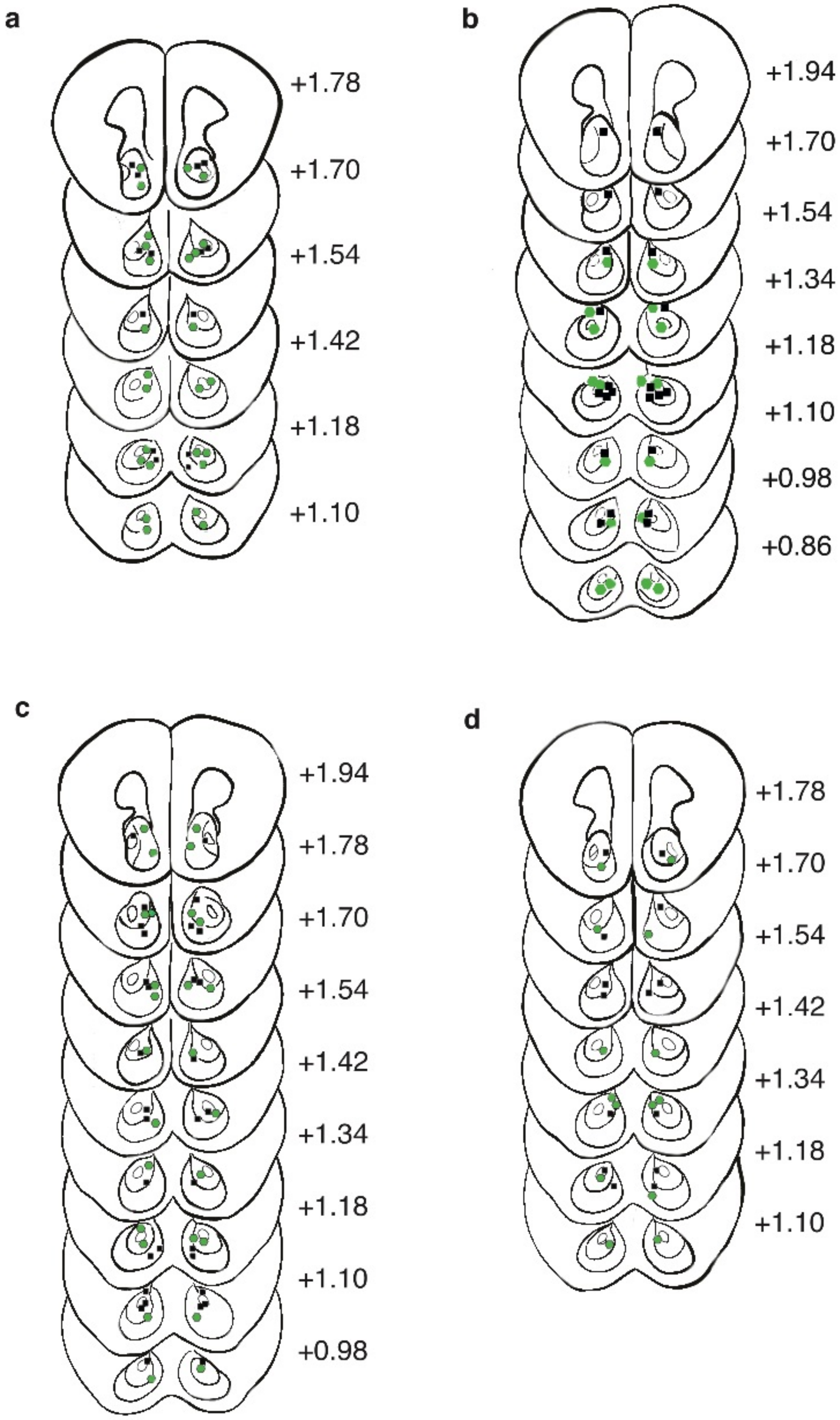
a
+1.78
+1.70
+1.54
+1.42
+1.18
+1.10
b
+1.94
+1.70
+1.54
+1.34
+1.18
+1.10
+0.98
+0.86
c
+1.94
+1.78
+1.70
+1.54
+1.42
+1.34
+1.18
+1.10
+0.98
d
+1.78
+1.70
+1.54
+1.42
+1.34
+1.18
+1.10

**Supplementary Figure 14. Schematic representation of injection sites for DREADDs experiments.** Each symbol represents the site of injection for one animal. **a.** Schematic representation of immediate post-training vehicle (black squares; n = 7) or CNO (green hexagons; n = 13) VS injection sites in the DREADDs experiment; **b.** Schematic representation of 120 min post-training vehicle (black squares; n = 11) or CNO (green hexagons; n = 9) VS injection sites in the DREADDs experiment; **c.** Schematic representation of post-training vehicle (black squares; n = 16) or CNO (green hexagons; n = 13) VS injection sites in sMWM experiment; **d.** Schematic representation of post-training vehicle (black squares; n = 7) or CNO (green hexagons; n = 7) VS injection sites before open field. Coordinates are expressed as mm from bregma.
