# Supplementary material for: Offline ventral subiculum-ventral striatum serial communication is required for spatial memory consolidation
Source: Nat Commun. 2019 Dec 16;10:5721. doi: 10.1038/s41467-019-13703-3 (PMC6915753; doi:10.1038/s41467-019-13703-3)
Supplement: Supplementary file 3 — Reporting Summary [file 41467_2019_13703_MOESM3_ESM.pdf]

nature research

Corresponding author(s): Andrea Mele

Reporting Summary

Nature Research wishes to improve the reproducibility of the work that we publish. This form provides structure for consistency and transparency in reporting. For further information on Nature Research policies, see Authors & Referees and the Editorial Policy Checklist.

Statistical parameters

When statistical analyses are reported, confirm that the following items are present in the relevant location (e.g. figure legend, table legend, main text, or Methods section).

n/a	Confirmed	
☐	☒	The exact sample size (*n*) for each experimental group/condition, given as a discrete number and unit of measurement
☐	☒	An indication of whether measurements were taken from distinct samples or whether the same sample was measured repeatedly
☐	☒	The statistical test(s) used AND whether they are one- or two-sided<br>*Only common tests should be described solely by name; describe more complex techniques in the Methods section.*
☐	☒	A description of all covariates tested
☐	☒	A description of any assumptions or corrections, such as tests of normality and adjustment for multiple comparisons
☐	☒	A full description of the statistics including central tendency (e.g. means) or other basic estimates (e.g. regression coefficient) AND variation (e.g. standard deviation) or associated estimates of uncertainty (e.g. confidence intervals)
☐	☒	For null hypothesis testing, the test statistic (e.g. *F*, *t*, *r*) with confidence intervals, effect sizes, degrees of freedom and *P* value noted<br>*Give P values as exact values whenever suitable.*
☒	☐	For Bayesian analysis, information on the choice of priors and Markov chain Monte Carlo settings
☒	☐	For hierarchical and complex designs, identification of the appropriate level for tests and full reporting of outcomes
☒	☐	Estimates of effect sizes (e.g. Cohen's *d*, Pearson's *r*), indicating how they were calculated
☐	☒	Clearly defined error bars<br>*State explicitly what error bars represent (e.g. SD, SE, CI)*

Our web collection on statistics for biologists may be useful.

Software and code

Policy information about availability of computer code

Data collection	Data were collected with readily available software (Ethovision 3.3; Timer 1.3; pClamp 10.7; Imaris 7.6.5; FIJI 1.0. NIH Image; Olympus Fluoview 4.2; NIS-Element Viewer; Anymaze 5.1; Debut Video Capture Software4.0)
Data analysis	Data were analyzed with Prism 5.0, Statview 5.0, and Statistica 7. Prism 5.0, MATLAB_R2017a and Adobe Illustrator CC 2015 were used in the production of data figures.

For manuscripts utilizing custom algorithms or software that are central to the research but not yet described in published literature, software must be made available to editors/reviewers upon request. We strongly encourage code deposition in a community repository (e.g. GitHub). See the Nature Research guidelines for submitting code & software for further information.

Data

Policy information about availability of data

All manuscripts must include a data availability statement. This statement should provide the following information, where applicable:

- Accession codes, unique identifiers, or web links for publicly available datasets
- A list of figures that have associated raw data
- A description of any restrictions on data availability

Provide your data availability statement here.

Field-specific reporting

Please select the best fit for your research. If you are not sure, read the appropriate sections before making your selection.

[x] Life sciences [ ] Behavioural & social sciences [ ] Ecological, evolutionary & environmental sciences

For a reference copy of the document with all sections, see nature.com/authors/policies/ReportingSummary-flat.pdf

Life sciences study design

All studies must disclose on these points even when the disclosure is negative.

Sample size	Sample size was not statistically predetermined, but based on our previous published data and common practice in these types of research studies.
Data exclusions	Pre-established exclusion criteria included lack of accurate stereotaxic targeting and/or viral expression, based on post-mortem brain tissue analyses or occlusion of both cannulae at the time of injection. A few mice (< 4%) were excluded based on aberrant behavior during training, before treatment. In the ODT a pre-established criterion required the mice to habituate to the objects during training before injection and testing. For the DREADDs experiment only mice showing the 70% of expression on the total VS surface were included in statistic.
Replication	Results were serially replicated through multiple cohorts.
Randomization	Mice were randomly assigned to experimental groups at the time of surgery, and all groups consisted of age-matched littermates.
Blinding	Data collection and analysis were not performed blind to the conditions of the experiment, except for dendritic spine density quantification and Fluoro-Gold/fos co-localization counting. All behavior experiments were controlled by computer systems, and data were collected and analyzed in an automated and unbiased way. The behavioral experiments were serially replicated by more than one investigator.

Reporting for specific materials, systems and methods

Materials & experimental systems

n/a	Involved in the study
[x]	[ ] Unique biological materials
[ ]	[x] Antibodies
[x]	[ ] Eukaryotic cell lines
[x]	[ ] Palaeontology
[ ]	[x] Animals and other organisms
[x]	[ ] Human research participants

Methods

n/a	Involved in the study
[x]	[ ] ChIP-seq
[x]	[ ] Flow cytometry
[x]	[ ] MRI-based neuroimaging

Antibodies

Antibodies used	fos (Santa Cruz Biotechnology, c-Fos (4): sc-52, cat.n. sc-52 ; lot K0915); Rhodamine (ImmunoResearch, Rhodamine RedTM-X-conjugated goat anti-rabbit, 111-295-144, lot 113658); HA (Cell Signaling Technology, HA-Tag (C29F4) Rabbit mAb #3724, lot 8); Vectashield Medium with Propidium Iodide (H-1300, Vector Labs).
Validation	All antibodies are validated for species by manufacturer.

Animals and other organisms

Policy information about studies involving animals; ARRIVE guidelines recommended for reporting animal research

Laboratory animals	CD1 male mice, 10-15 weeks old were used in the experiment.<br>Details can be found in the method section Supplementary material page 2 first paragraph.
Wild animals	Study did not involve wild animals.
Field-collected samples	Study did not involve field collected samples.

April 2018
